# Pain Assessment and Treatment in Dementia at the Time of Coronavirus Disease COVID-19

**DOI:** 10.3389/fneur.2020.00890

**Published:** 2020-08-26

**Authors:** Damiana Scuteri, Marta Matamala-Gomez, Sara Bottiroli, Maria Tiziana Corasaniti, Roberto De Icco, Giacinto Bagetta, Paolo Tonin

**Affiliations:** ^1^Pharmacotechnology Documentation and Transfer Unit, Preclinical and Translational Pharmacology, Department of Pharmacy, Health and Nutritional Sciences, University of Calabria, Rende, Italy; ^2^“Riccardo Massa” Department of Human Sciences for Education, University of Milano-Bicocca, Milan, Italy; ^3^Giustino Fortunato University, Benevento, Italy; ^4^IRCCS Mondino Foundation, Pavia, Italy; ^5^Department of Health Sciences, University “Magna Graecia” of Catanzaro, Catanzaro, Italy; ^6^School of Hospital Pharmacy, University “Magna Graecia” of Catanzaro, Catanzaro, Italy; ^7^Neurorehabilitation Unit, IRCCS Mondino Foundation, Pavia, Italy; ^8^Department of Brain and Behavioral Sciences, University of Pavia, Pavia, Italy; ^9^Regional Center for Serious Brain Injuries, S. Anna Institute, Crotone, Italy

**Keywords:** pain, dementia, COVID-19, assessment, telemedicine

## Introduction

The infection of severe acute respiratory syndrome coronavirus (SARS-CoV) 2 has raised rapidly from the outbreak in Wuhan within the Chinese Hubei province all over the world resulting in a pandemic emergency, which has remarkably affected the Italian population since February 21, 2020 (1). COVID-19 (COrona VIrus Disease 2019) presents the highest rate of severity and mortality in the elderly, characterized by several comorbidities contributing to a worse prognosis (2). This is exacerbated by the circulation and spread in long-term care facilities (3). Among the concurrent chronic conditions affecting the aged patients and the oldest old, one of the most frequent is represented by cognitive impairment in dementia, known as Alzheimer‘s disease and related dementias (ADRD) (4). It is known that age represents a highest risk factor for pain and dementia (5). In addition, about half of the people suffering with dementia experience regular pain (6). Pain can be encountered in different types of dementia, such as Alzheimer's disease (AD), vascular dementia (VaD), fronto-temporal dementia (FTD), and Parkinson's disease (PD), and it could appear in different forms (e.g., nociceptive pain, neuropathic pain, and central pain) (5). Importantly, the occurrence of pain in dementia could lead to further complications in the patients' healthcare routine. At this moment, due to the COVID-19 emergency, a large amount of old people presenting dementia and pain cannot attend to the hospital to receive their usual healthcare routine to manage pain. In this regard, the introduction of new digital technologies in the field of medicine—commonly known as “telemedicine” or “telehealth” (7)—can pave the way for treating pain in patients with dementia from the comfort of their own home (8).

## Dementia, Pain, and COVID-19

ADRD affect some 50 million people worldwide (9) and 900–1,000 per 100,000 inhabitants in Italy (10). The 12% of COVID-19 positive dead patients in Italy suffered from dementia (11), and 43% of deaths occurs in the oldest old (12). Apart from being aged, demented patients may have difficulties to remember preventative measures, thus resulting in a higher risk of infection, even more in nursing-home residents (4). Moreover, the mental and cognitive health of demented patients can be worsened by COVID-19. These patients suffer from several behavioral symptoms, like agitation and aggression, known as behavioral and psychological symptoms of dementia (BPSD), which can be enhanced by social distancing (13). A greater concern is for patients in need of hospitalization for COVID-19, since a new environment is proven to increase BPSD (14). Losing face-to-face contact with people familiar to the patients can bear a remarkable burden (4), in terms both of anxiety and of cognition. Moreover, COVID-19 induces delirium due to hypoxia, which can exacerbate dementia (4). Cognitive deterioration is common in course of acute respiratory distress syndrome (ARDS) and it can last also in the long term, complicating several aspects, such as memory and attention (15, 16). In particular, COVID-19 seems to be associated with neurologic manifestations as confusion (9%), dizziness (17%), impairment of consciousness (8%), risk of stroke (3%), anosmia (6%), hypogeusia (6%), and ataxia (1%) (17). Moreover, neuropathies can also occur (16). This issue can play a pivotal role in patients affected by ADRD, since they often present mixed pain states like osteoarthrosis and diabetic neuropathy, due to their advanced age (18). Mobility, already impaired by these conditions, can result in being very difficult to recover after hospitalization, mainly in intensive care units (ICU). The issue of worsened conditions is even more worrying in this period in which follow-up and accurate review of therapy against BPSD are postponed in order to reduce the risk of contagion (13).

In this field, pain is considered one of the most important causes of BPSD (19). In particular, the BPSD can arise as a result of pain through agitation or aggression, representing a stressful factor for both the patients and the caregivers (6). Another important issue is the impact of neuropathological changes occurring in dementia, which could affect patients' pain perception (20). Concerning this, it is known that in patients with ADRD the neuropathological changes occurring after the onset of the clinical condition have a greater impact in the medial pain system than on the lateral pain system (20). This means that in patients presenting ADRD, there is a higher impairment of the cognitive-evaluative and motivational-affective aspects of pain than in sensory-discriminative ones (20). However, in patients with VaD, lesions in white matter lead to several disconnections between brain areas in a neurobiological process known as “deafferentation” and provokes an increase in the motivational-affective aspects of pain (6). This type of pain—commonly known as “central neuropathic pain” —has also been shown in patients with stroke (21), and with VaD (22, 23). Nevertheless, in FTD patients the atrophy in the prefrontal cortex can lead to a decrease in the motivational-affective aspects of pain, similarly to those presenting ADRD (24). Overall, the alterations in both the afferent transmission pathways and the endogenous descending inhibitory transmission control systems lead to an altered pain processing in patients with dementia (25). Moreover, it has been shown that the more severe the cognitive impairment, the bigger the difference in pain experience between demented and non-demented populations (5).

## Pain Assessment and Neuro-Rehabilitation: the Contribution of Technology at the Time of COVID-19

The 72% of patients older than 85 years suffer from pain (26, 27), and this amount can reach the 80% for nursing-home guests with ADRD (6) and definitely increase in ARDS and intubation. Pain diagnosis and assessment through self-report represents the gold standard, but it cannot be applied in patients with severe ADRD because of their limited communication skills (28). In these patients, underdiagnosed pain may induce BPSD like agitation (29, 30), requiring the use of neuroleptics increasing cardio cerebrovascular accidents (31) and, hence, predisposing to increased risk in course of COVID-19. In this situation, the ABCDEF bundle can be recommended: assess, prevent and manage pain; both spontaneous awakening and breathing trials; choice of sedation; delirium monitoring and management; early mobility and exercise; and family engagement and empowerment (16, 32). Although telemedicine can be not suitable to provide virtual neurologic examination (13), it can be very useful to manage BPSD (13) and pain (33). It can indeed represent an important option to provide accurate treatment also with drugs like opioids endowed with serious adverse reactions (34), including immune system, and thus involved in COVID-19 management (33). Therefore, the assessment of pain is fundamental to improve the quality of life and reduce the risk of death of demented patients, even more in this difficult scenario. For patients with severe dementia observational assessment tools can be applied. In particular, the Mobilization–Observation–Behavior–Intensity–Dementia (MOBID)-2 pain scale that allows the caregiver to rate the intensity of both the musculoskeletal pain, through the observation of pain behavioral indicators (pain noises, facial mimics, and defense moves) during the execution of five guided movements to unravel also hidden conditions, and the visceral pain (35). Furthermore, some reviews highlighted that the same motor rehabilitative treatment, delivered from afar or face to face, produces the same results, suggesting that telerehabilitation is not inferior in comparison with in-person therapy (36, 37). In this situation, motor telerehabilitation can be very useful to improve motor activity, according to the ABCDEF bundle, and tele-care may also allow to establish a safe contact with the caregiver whom can be instructed in streaming by the health assistant (38). The use of mask may prevent the assessment of facial expressions. Moreover, another assessment test for intubated patients, with specific non-verbal pain scales examined in ICU, is the Critical-Care Pain Observation Tool (CPOT) (39). This pain scale allows to observe pain also in the presence of the endotracheal tube and to evaluate the compliance with the ventilator, and it has proven to have good validity, reliability, feasibility, and clinical utility (39–41). The main features of the proposed pain assessment tools are reported in Table 1.

**Table 1:** Characteristics of the pain assessment tools useful for non-communicative patients with severe dementia and intubated.

**Pain assessment tool**	**Authors and year of first publication**	**Type of scale**	**Number of items**	**Time of execution**	**Qualification of rater**	**Validity and reliability**
Mobilization–Observation–Behavior–Intensity–Dementia (MOBID)-2.	(35)	Observational scale.	It consists of two parts of 5 items each. Part 1: assessment of musculoskeletal pain observing pain behavior during the execution of five guided movements. Part II: assessment of pain from internal organs, head and skin pain behavioral indicators, and localization of pain crossing on pain drawing.	Time-efficient in use (mean 4.37 min, range 2.0–7.0).	Trained nurse.	Moderate to excellent agreement was demonstrated for behaviors and pain drawings (κ = 0.41–0.90 and κ = 0.46–0.93). Inter-rater and test–retest reliability for pain intensity: ICC 0.80–0.94 and 0.60–0.94. Internal consistency: Cronbach's α ranging 0.82–0.84. Good face-, construct- and concurrent validity. Correlation of overall pain intensity with physicians' clinical examination and defined pain variables (rho = 0.41–0.64).
Critical-Care Pain Observation Tool (CPOT).	(39)	Observer rated scale.	It consists of 4 items: facial expression, body movement, ventilator compliance, and muscle tension.	The patient is observed for 1 min at rest and during and after nociceptive procedure.	Trained nurse.	Inter-rater reliability: *k* = 0.52–0.88. Acceptable reliability and validity, with significant discriminant validity (paired *t*-tests, *P* ≤ 0.001). Criterion validity: analyses of variance ANOVA (*P* ≤ 0.001) and Spearman correlations (0.40–0.59, *P* ≤ 0.001).

Interestingly, previous investigations have described the use of telemedicine as a useful tool to follow or treat clinical populations in catastrophic situations or in public health emergencies (42). Through telemedicine systems, patients can be efficiently screened, and this could represent an effective approach in the current worldwide emergency of COVID-19. By using telemedicine systems, it is also possible to protect patients, clinicians, and the community from virus exposure (8). Moreover, telemedicine systems allow physicians and patients to be in contact anytime (24/7) through smartphones, tablet, or webcam enabled computers (8) and tackle some clinical issues related to expenses, prevalence, and other treatment barriers associated with the patients' management. In particular, telemedicine has been used for pain assessment through digital diaries or personal digital assistants (43, 44), to provide an accurate and easy monitoring of pain symptoms. Regarding treatment delivery in pain patients, novel telemedicine strategies have been found effective to facilitate consultation and talk therapy and to provide rehabilitation pain trainings (45–49). For instance, telemedicine systems have been proposed to provide behavioral medicine interventions in chronic pain patients through a self-regulation training targeting both the sensory and affective components of pain (50). In addition, training programs through video-conferencing have been also used for pain treatments (46, 50).

## Discussion

The SARS-CoV 2 has changed the management of chronic conditions often occurring in the main target of COVID-19 represented by the aged population. One of the most common comorbidities in these patients is dementia, often accompanied by chronic pain. The assessment and management of pain in demented patients is necessary during COVID-19 pandemic emergency, and the use of telemedicine can allow a safe handling reducing the access to hospitals and clinics to contain contagion. We suggest pain management to improve the quality of life of patients and to reduce agitation (51): accurate review of analgesic and antipsychotic therapy of BPSD can reduce cardiocerebrovascular events, an important risk factor for bad prognosis of COVID 19. Pain is often misunderstood and undertreated; therefore, educational programs for physicians and caregivers are needed (52, 53) to improve “pre-habilitation,” the process of optimizing general health fundamental to cope better with the stress condition (16), and neurorehabilitation of demented patients after COVID 19. Furthermore, novel telemedicine systems should be also taken in consideration to provide assessment and rehabilitation pain trainings to improve neurorehabilitation of patients suffering from dementia in the new era of COVID-19.

## Author Contributions

DS, MC, GB, and PT have conceived the work. All authors listed have made a substantial, direct and intellectual contribution to the work, and approved it for publication.

## Conflict of Interest

The authors declare that the research was conducted in the absence of any commercial or financial relationships that could be construed as a potential conflict of interest.
